# Rapid endothermal development of juvenile pacific bluefin tuna

**DOI:** 10.3389/fphys.2022.968468

**Published:** 2022-08-19

**Authors:** Takashi Kitagawa, Takaaki K. Abe, Keitaro Kubo, Ko Fujioka, Hiromu Fukuda, Yosuke Tanaka

**Affiliations:** ^1^ Atmosphere and Ocean Research Institute, University of Tokyo, Tokyo, Japan; ^2^ Fisheries Resources Institute, Japan Fisheries Research and Education Agency, Shizuoka, Japan; ^3^ Fisheries Resources Institute, Japan Fisheries Research and Education Agency, Yokohama, Japan

**Keywords:** Pacific bluefin tuna, body temperature, physio-logging, whole-body heat-transfer coefficient, heat production rate, adaptation, thunnus

## Abstract

An important trait of Pacific bluefin tuna (PBT) is their ability to maintain their body temperature above the ambient temperature, which allows them to occupy a wider ecological niche. However, the size at which this ability in nature develops is unclear. Therefore, this study aimed to clarify this point by monitoring the body temperature and the surrounding ambient temperature as the fish grew. PBT with fork lengths (FLs) ranging from 19.5 to 28.0 cm were implanted with archival electronic tags and released into the ocean. Data from 41 fish were obtained (recorded body and water temperatures, light level, and swimming depth (pressure) at 30-s intervals) and analyzed to elucidate the development of the ability of PBT to maintain a high body temperature. Body temperature of a PBT (< FL of ca. 40 cm) decreased in response to a vertical movement down to cooler depths, but higher body temperatures were maintained as the fish grew. The body temperature was then continuously maintained above ambient temperatures and fluctuated independently when fish attained more than 40 cm FL. Estimation of the whole-body heat-transfer coefficient and heat-production rate indicated that the latter decreased slowly with growth, while the former decreased by one order of magnitude when tuna reached 52 cm FL. Additionally, in the daytime, the whole-body heat-transfer coefficient was significantly higher than that at nighttime. Unlike other fishes including other *Thunnus* species, inhabiting tropical/subtropical waters, PBT rapidly acquire higher thermo-conservation ability when young, allowing capture of high-quality prey abundant in temperate waters to support high growth rates during early life.

## 1 Introduction

**FIGURE 1:**
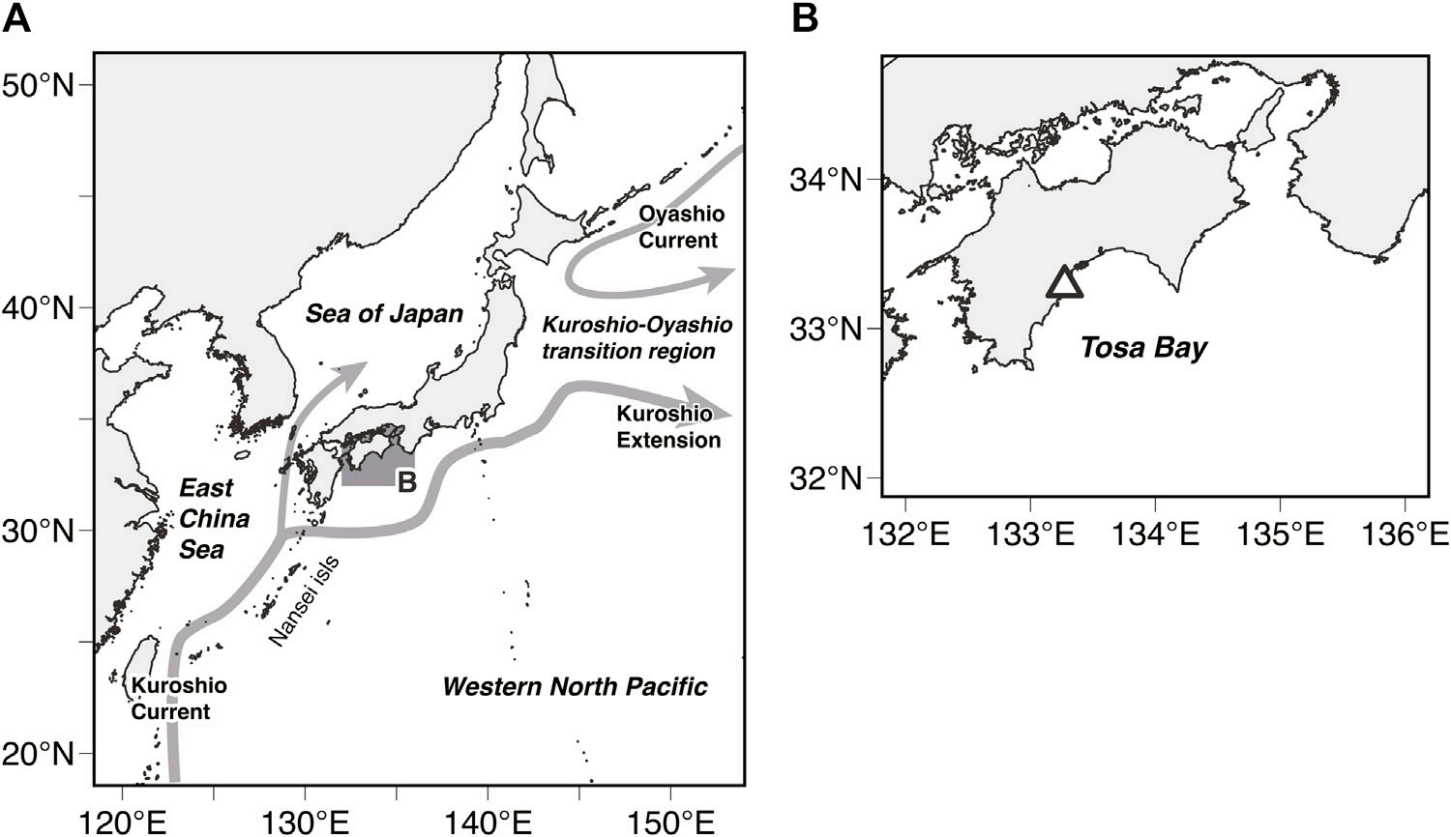
**(A)** Map of the western North Pacific Ocean showing the study area (shaded area). Schematic of the near-surface currents around Japan: Kuroshio Current, Kuroshio Extension, and Oyashio Current (gray arrows) **(B)** The white triangle represents the release location of tagged Pacific Bluefin Tuna (PBT).

Pacific bluefin tuna (PBT; *Thunnus orientalis*) is an important commercial fisheries species owing to its excellent meat quality and economic importance. The PBT breed from April to June between the Philippines and the Ryukyu (Nansei) Islands of Japan and during August in the Sea of Japan (Okiyama, 1974; Chen et al., 2006; Tanaka et al., 2007) and a third breeding ground has been reported (Tanaka et al., 2019). They are then transported by sea currents to Japanese coastal areas in the summer (e.g., by the Kuroshio Current) 60–90 days after hatching (Chen et al.*,* 2006; Tanaka et al., 2006; Satoh et al., 2008; Kitagawa et al., 2010; Satoh, 2010). While some PBT remain in the coastal waters around Japan, others migrate from the Kuroshio-Oyashio transition region (Figure 1) to the eastern Pacific (Orange and Fink, 1963; Clemens and Fittner, 1969; Bayliff, 1980; Bayliff et al., 1991; Inagake et al., 2001; Itoh et al., 2003; Kitagawa et al., 2009; Fujioka et al., 2018). Finally, after spending several years in the eastern Pacific, they return to their breeding grounds (Bayliff, 1980; Block, 2005; Boustany et al., 2010).

PBT are widely distributed in the Pacific because, like other tuna (Scombridae, *Thunnini*), they can elevate the temperature of their locomotor muscle, viscera, brain, and eye tissues above that of the water (*T*_*a*_) (Carey and Teal, 1966; Carey et al., 1971; Graham, 1975; Carey, 1981; Brill, 1996; Altringham and Block, 1997; Dickson and Graham, 2004). By raising their body temperature (*T*_*b*_) in this way, these fish species have expanded their niches to low-temperature waters and improved their ability to sustain high-speed swimming due to their high aerobic metabolism (Carey, 1973; Carey, 1981; Stevens and Carey, 1981; Carey et al., 1984; Block and Finnerty, 1994; Brill, 1996; Dickson and Graham, 2004). As a result, they can use the marine environment three-dimensionally and can occupy a larger area of the marine ecosystem (Watanabe et al., 2015). Among the species with this trait, tuna have long been studied for their ability to maintain *T*_*b*_ because they have a large proportion of red muscle suitable for continuous swimming (Carey, 1973; Dickson, 1996) and have developed a mechanism for maintaining *T*_*b*_.

The tuna’s ability to maintain *T*_*b*_ is associated with the development of the rete mirabile tissue (Kishinouye, 1923), a reticulated complex in which arteries and veins are arranged alternately. Large retia are found in the blood vessels supplying the swimming muscles (Kishinouye, 1923) and certain visceral organs (Eschricht and Müller, 1835; Gibbs and Collette 1967; Carey et al., 1984; Fudge and Stevens, 1996; Whitlock et al., 2013; Bernal et al., 2017) and play a role in preventing the loss of heat generated by the red muscles (Carey et al., 1971; Carey and Gibson, 1983). In particular, the rete is well developed in bluefin tuna species, including the Atlantic bluefin tuna (*T. thynnus*; ABT), southern bluefin tuna (*T. maccoyii*; SBT), and PBT (Sharp and Dizon, 1978; Holland et al., 1992). However, during the larval and juvenile stages, these species cannot maintain *T*_*b*_; rather, the fish gradually gains the ability to maintain *T*_*b*_ by developing a rete mirabile as it grows.

Regarding the development of the ability to maintain *T*_*b*_, PBT with a fork length (FL) of ≥30 cm immediately after capture have been found that 3–4°C of the thermal excess (*T*_*b*_–*T*_*a*_.). This suggests that PBT of this size and larger have a developed rete mirabile, enabling them to maintain *T*_*b*_ (Funakoshi et al., 1985). Kubo et al. (2008) measured ‘steady-state’ *T*_*b*_ in PBT and found that the thermal excess increased from 0.1 to 0.8°C in 16.5–32.6 cm FL to 2.6–4.4°C in 54.5–55.5 cm FL. As the FL increases in this species during this stage, the length of the lateral net, the number of blood vessels, and the cross-sectional area of the visceral net rapidly increase (Malik et al., 2020). Total red muscle metabolic heat production capacity also increases with fish size because of the increase in total red muscle mass, thereby contributing to the increasing thermal excess (Malik et al., 2021). Furthermore, at approximately 25 cm FL, the dietary habits of PBT change significantly. The trophic level increases by one stage and the caudal fin morphs into a shape more suitable for swimming; therefore, it is suspected that the ability to maintain *T*_*b*_ develops at approximately 25 cm FL (Shimose et al., 2013; Kitagawa and Fujioka, 2017). Meanwhile, the long-term measurement of *T*_*b*_, *T*_*a*_, and swimming depth using biologging revealed that individuals with ≥45 cm FL have already developed their ability to maintain *T*_*b*_ (e.g., Kitagawa et al., 2000; Kitagawa et al., 2001; Kitagawa et al., 2006; Kitagawa et al., 2007a; Kitagawa et al., 2007b; Whitlock et al., 2013; Fujioka et al., 2018). However, owing to technical constraints, detailed information about the *T*_*b*_ of free-swimming in nature juvenile PBT (especially <30 cm FL), the timing of PBT developing their ability to maintain *T*_*b*_, and the details of its mechanism remain unknown. This information would help to elucidate the adaptation to and clarify the energy budget for juvenile growth in cooler temperate waters.

The microtechnology of biologging is progressing rapidly and current electronic devices are much smaller than those used previously (Kitagawa et al., 2000; Kitagawa, 2013), being one twenty-fifth of the size (Kitagawa et al., 2019), and they can be used for smaller-sized PBT (Furukawa et al., 2017). Therefore, in this study, the development of the ability of PBT to maintain *T*_*b*_ and its exact timing were determined by attaching electronic devices (archival tags) to small-sized PBT immediately after their arrival on the coast of Japan, releasing them, and analyzing the collected *T*_*b*_ and *T*_*a*_ data (i.e., physio-logging data). We note that Furukawa et al. (2017) and Fujioka et al. (2018) analyzed part of the same data used in the present study, reporting the vertical and horizontal movements of PBT in relation to seasonal and geographical changes in *T*_*a*_.

## 2 Materials and methods

### 2.1 Summary of analysis data and electronic devices

In this study, data were obtained from archival tags (LAT2910; Lotek Wireless Inc. Ontario, Canada) attachment and release survey conducted on PBT by the National Institution of Far Seas Fisheries, Fisheries Research Agency (present name: Japan Fisheries Research and Education Agency Fisheries Resources Institute) in Tosa Bay, Kochi Prefecture (Furukawa et al., 2017; Fujioka et al., 2018) from July to August in 2012, 2013, 2014, and 2015. Of the total 3,281 fish (1,044, 2012; 1,725, 2013; 236, 2014, and 276, 2015) captured by trawling, 406 (75, 2012; 62, 2013; 77, 2014, and 107, 2015) were implanted with an archival tag and released into the same area. After release, of the 100 fish (23, 2012; 8, 2013; 23, 2014, and 39, 2015) that were recaptured off Tosa Bay and its adjacent waters and in California in the United States, data from 41 (5, 2012; 1, 2013; 5, 2014, and 30, 2015) for which *T*_*a*_, *T*_*b*_, depth, and light level could be recorded were used for analysis. The tagging procedure is described in detail in Furukawa et al. (2017) and Fujioka et al. (2018). Fish size was determined by straight fork length (FL).

The archival tags consisted of a body (18 mm × 8.5 mm) and stalk (154 mm), weighing 3.3 and 1.2 g in air and water, respectively. A temperature sensor and an illuminance sensor were attached to the tip of the stalk, and another temperature sensor and a pressure sensor were attached to the main body. The temperature sensor had a resolution of 0.02°C in the range of –5–45°C, while the depth sensor had a maximum measurable depth of 500 m, and resolution set to 0.25 m. These four sensors were set to measure and record *T*_*a*_, the light level, *T*_*b*_ in the peritoneal cavity (where the tag was attached), and depth every 30 s (Furukawa et al., 2017).

### 2.2 Data analysis

#### 2.2.1 Time-series data analysis

Igor Pro eight software (WaveMetrics Inc., Portland, OR, United States) was used to analyze the water temperature, body temperature, depth, and light level data recorded at 30-s intervals by the archival tags. The “Ethographer” package (Sakamoto et al., 2009) in Igor Pro eight was introduced to analyze the time-series data by time-series and Mask analyses. A significant error occurred when the sunrise and sunset times were estimated based on the light level data. Therefore, the sunrise and sunset times near Tosa Bay, given by the National Astronomical Observatory of Japan were used to determine day and night. Because short-term data-measurement abnormalities were observed in some individuals, these data were excluded from the analysis (14.1% of all data points). Temporary measurement abnormalities were corrected by taking a moving average of the values before and after the abnormal point (0.003% of all data points).

#### 2.2.2 FL and weight estimation during swimming

To clarify the development of the ability of PBT to maintain T_b_, the FL was used as an index of growth (Funakoshi et al., 1985; Kitagawa and Fujioka, 2017). The growth rate of PBT is constant, at 0.45 cm per day at 30–120 days of age (Jusup et al., 2011), and is not well adapted to the von Bertalanffy growth function widely used as a growth formula for fish (Fukuda et al., 2015). Therefore, the change in FL during the swimming period was estimated by linearly regressing FL from the time of release to recapture. For fish with no FL data at the time of recapture, FL was estimated from the average growth rate calculated from the linear regression equation of other fish. Body weight during the swimming period was estimated using the weight relationship formula (body weight = 4.85 × 10^−3^ FL^3.39^) used in previous studies on PBT with 18.5–62.5 cm FL (Malik et al., 2020).

#### 2.2.3 Heat budget model

Generally, changes in body temperature can be represented as follows using the heat balance model from Schmidt-Nielsen (1997):

Time change in body temperature = heat loss + heat production.

This relationship is described in Eq. 1 using *T*_*a*_ and *T*_*b*_ (Kitagawa et al., 2001).

$$\frac{dT_{b}}{dt}=\lambda \left(T_{a}-T_{b}\right)+\frac{dT_{m}}{dt},$$ (1)

where *dT*_*b*_ */dt* indicates the time change in body temperature (°C·s^−1^), *λ* indicates the whole-body heat-transfer coefficient (s^−1^), and *dT*_*m*_ */dt* indicates the heat production rate (°C·s^−1^). The first term on the right side indicates heat loss due to heat conduction, and a lower whole-body heat-transfer coefficient corresponds to a greater heat insulation property. The second term represents heat production. When Eq. 1 is converted to a different format,

$$\frac{T_{b\left(n+1\right)}-T_{b\left(n\right)}}{\Delta t}=\lambda \left(T_{a\left(n\right)}-T_{b\left(n\right)}\right)+\frac{dT_{m}}{dt},$$ (2)

where *n* represents the number of data points. To estimate the whole-body heat-transfer coefficient *λ* and the heat production rate *dT*_*m*_/*dt* from the body and water temperature data, Eq. 2 was converted into a data string vector format of n–1 for the time-series body and water temperature data with the number of data points *n*, as follows:

$$\left(\begin{array}{c} T_{b\left(n\right)}-T_{b\left(n-1\right)}\\ \vdots \\ T_{b\left(2\right)}-T_{b\left(1\right)} \end{array}\right)=\Delta t\cdot \left(\begin{array}{cc} T_{a\left(n-1\right)}-T_{b\left(n-1\right)} & 1\\ \vdots & \vdots \\ T_{a\left(1\right)}-T_{b\left(1\right)} & 1 \end{array}\right)\left(\begin{array}{c} \lambda \\ dT_{m}/dt \end{array}\right).$$ (3)

If the following is assumed:

$$y=\left(\begin{array}{c} T_{b\left(n\right)}-T_{b\left(n-1\right)}\\ \vdots \\ T_{b\left(2\right)}-T_{b\left(1\right)} \end{array}\right),$$

$$A=\left(\begin{array}{cc} T_{a\left(n-1\right)}-T_{b\left(n-1\right)} & 1\\ \vdots & \vdots \\ T_{a\left(1\right)}-T_{b\left(1\right)} & 1 \end{array}\right),$$

$$x=\Delta t\cdot \left(\begin{array}{c} \lambda \\ dT_{m}/dt \end{array}\right),$$

then Eq. 3 would be

y=A⋅x, (4)

which is a linear model (e.g. Boyd and Vandenberghe, 2018). By transforming Eq. 4 into the following Eq. 5, *λ* and *dT*_*m*_/*dt* can be estimated:

$$x={\left(t^{A}\cdot A\right)}^{-1}\cdot t^{A}\cdot y,$$ (5)

where ^***t***^ ***A*** represents the transposed matrix of ***A***. The vertical movement frequency of PBT differs between day and night, and *T*_*b*_ is higher in daytime than in nighttime (Kitagawa et al., 2001); therefore, Eq. 3 was divided into day and night, and *λ* and *dT*_*m*_ */dt* were obtained.

#### 2.2.4 Statistical analysis

The relationships between the response (*λ* and *dT*_*m*_ */dt*) and the explanatory variables (FL and Day/Night) were evaluated using generalized additive mixed models (GAMMs), which allow for nonlinear relationships between parameters. The models were estimated using the *gam* function in the “mgcv” package of R v4.2.0 (Foundation for Statistical Computing, Vienna, Austria), and the random intercept was added to account for the differences between individuals (i.e., differences in the insertion position of the electronic tags or the development state of the rete mirabile between individuals). Model selection was conducted by comparing the Akaike information criterion (AIC) and Bayesian information criterion (BIC).

From the time-series data of the whole-body heat-transfer coefficient and the heat production rate, we explored the range of body sizes (FL) over which PBT rapidly developed the ability to maintain *T*_*b*_. Segment regression analysis was performed to identify the end point of this rapid development. First, the parameters were log_10_-transformed because they decreased exponentially with FL. Next, the relationships between the log_10_-transformed parameters and FL were modeled using a linear mixed-effect (lme) model with the *lme* function in the “nlme” package of R. The random effect caused by the individual differences was modeled as the random intercept. Segment regressions were performed for these lme models, comparing AIC (and also BIC) to determine if the breakpoints and inflection points should be set. The *segmented* function in the “segmented” package was used for this analysis.

## 3 Results

The average FL of the 41 fish used in the analysis was 23.7 cm (range: 19.5–28.0 cm) and 46.2 cm (range: 25.0–210.3 cm) for recapture ed individuals (Table 1). The interval from release to recapture was 4–277 days. Depending on the individual, the data for 24–48 h after release and for 24 h before recapture showed the influence of handling and data loss; hence, they were not used in the analysis.

**TABLE 1:** Information regarding the deployed individuals. FL, straight fork length. Values in parentheses at recapture represent estimated fork length at recapture. Details are given in Furukawa et al. (2017) and Fujioka et al. (2018).

Fish ID	Capture date	FL at capture (cm)	Recapture date	FL at recapture	FL range for analysis	Data length
2012-0925	Jul-28-2012	24.0	Aug-25-2014	(60.5 cm)	24.2–60.5	148 days
2012-0932	Jul-29-2012	28.0	Dec-19-2012	53.5 cm	29.1–47.2	102 days
2012-0948	Jul-29-2012	24.5	Oct-21-2012	50.0 cm	26–49.8	79 days
2012-1127	Aug-10-2012	26.5	Jul-28-2013	(97.8 cm)	29.7–97.8	277 days
2012-1128	Aug-10-2012	27.0	Aug-03-2014	(122.9 cm)	119.9–122.9	12 days
2013-1766	Aug-15-2013	23.0	Sep-19-2014	(89.9 cm)	23.5–89.9	270 days
2014-2880	Aug-14-2014	21.5	Nov-18-2014	47.2 cm	23.4–47.1	89 days
2014-2914	Aug-18-2014	20.5	Nov-17-2014	(42.7 cm)	21.2–42.7	88 days
2014-2922	Aug-18-2014	23.0	Nov-13-2014	49.0 cm	23.9–49.1	84 days
2014-2940	Aug-20-2014	23.5	Nov-08-2014	40.0 cm	23.7–39.7	78 days
2014-2952	Aug-20-2014	25.0	Nov-13-2014	46.5 cm	25.3–46.6	84 days
2015-2850	Jul-22-2015	23.0	Aug-21-2015	(30.1 cm)	24.2–30.1	24 days
2015-2880	Jul-22-2015	22.5	Aug-22-2015	30.0 cm	23.7–30	26 days
2015-2914	Jul-23-2015	22.0	Sep-10-2015	32.4 cm	22.8–32.2	44 days
2015-2915	Jul-27-2015	22.5	Sep-18-2015	37.7 cm	27.1–37.7	37 days
2015-2961	Jul-25-2015	23.5	Sep-02-2015	28.9 cm	26–28.9	21 days
2015-2962	Jul-25-2015	21.5	Aug-31-2015	29.8 cm	23.1–29.8	30 days
2015-2975	Jul-25-2015	23.0	Aug-29-2015	32.1 cm	24.8–32.1	28 days
2015-2977	Jul-25-2015	19.5	Sep-04-2015	30.3 cm	21.3–30.3	34 days
2015-2979	Jul-25-2015	22.0	Sep-01-2015	25.0 cm	23.4–24.9	19 days
2015-2997	Jul-27-2015	23.0	Aug-19-2015	(28.6 cm)	26.9–28.6	7 days
2015-3001	Jul-28-2015	23.5	Sep-06-2015	33.7 cm	27.3–33.6	25 days
2015-3008	Jul-28-2015	25.0	Aug-31-2015	27.4 cm	26–27.4	19 days
2015-3037	Jul-29-2015	23.0	Aug-27-2015	30.0 cm	26.4–30	15 days
2015-3763	Jul-30-2015	24.5	Aug-31-2015	33.0 cm	28–33	19 days
2015-3769	Aug-01-2015	25.0	Aug-28-2015	(31.6 cm)	27.7–31.6	16 days
2015-3778	Aug-18-2015	24.0	Sep-15-2015	30.3 cm	24.7–30.3	25 days
2015-3783	Aug-02-2015	26.0	Aug-22-2015	(30.5 cm)	28.5–30.5	8 days
2015-3787	Aug-02-2015	25.5	Aug-16-2015	31.0 cm	29.4–31	4 days
2015-3789	Aug-02-2015	26.0	Aug-16-2015	29.5 cm	28.5–29.5	4 days
2015-3792	Aug-02-2015	26.5	Oct-21-2015	47.2 cm	29.1–47.1	70 days
2015-3793	Aug-02-2015	24.0	Sep-11-2015	31.4 cm	25.9–31.5	30 days
2015-3798	Aug-05-2015	21.0	Aug-29-2015	25.9 cm	22.6–24.5	9 days
2015-3799	Aug-05-2015	21.0	Aug-31-2015	35.0 cm	24.8–35	19 days
2015-3808	Aug-06-2015	21.5	Aug-28-2015	30.0 cm	24.2–29.6	14 days
2015-3809	Aug-06-2015	21.0	Sep-08-2015	30.6 cm	22.7–30.6	27 days
2015-3978	Aug-18-2015	27.0	Sep-09-2015	29.7 cm	27.4–29.7	19 days
2015-3984	Aug-13-2015	23.0	Sep-03-2015	28.8 cm	23.8–28.8	18 days
2015-3985	Aug-04-2015	23.0	Sep-04-2015	27.3 cm	24.1–27.2	22 days
2015-3989	Aug-18-2015	24.0	Aug-29-2015	26.0 cm	24.5–26	8 days
2015-3992	Aug-19-2015	27.0	Sep-08-2015	34.0 cm	27.7–34	18 days

### 3.1 Development of pacific bluefin tuna ability to maintain *T*_*b*_

**FIGURE 2:**
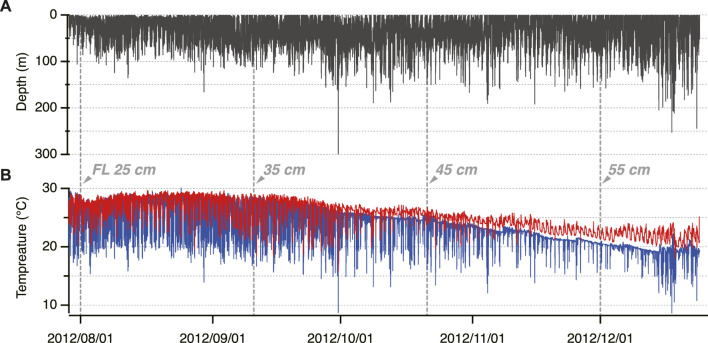
Example of the time-series data of electronically tagged Pacific Bluefin Tuna (ID 2012-0925). **(A)** Swimming depth **(B)** Body temperature (red) and ambient water temperature (blue).

Time-series data of the depth, *T*_*b*_, and *T*_*a*_ for 148 days are shown by a representative example of one individual released and recaptured in 2012 (ID 2012-0925) (Figure 2). During this period, its FL increased from 24.2 to 60.5 cm. The PBT (24.2–39.8 cm FL) made frequent vertical movements to a maximum of 342.3 m from August to September. *T*_*b*_ decreased in response to this vertical movement but was eventually maintained at higher temperatures as the fish grew. Around October (39.8–47.4 cm FL), *T*_*b*_ was continuously maintained above *T*_*a*_ (Figure 2B); it fluctuated independently and increased in daytime.

**FIGURE 3:**
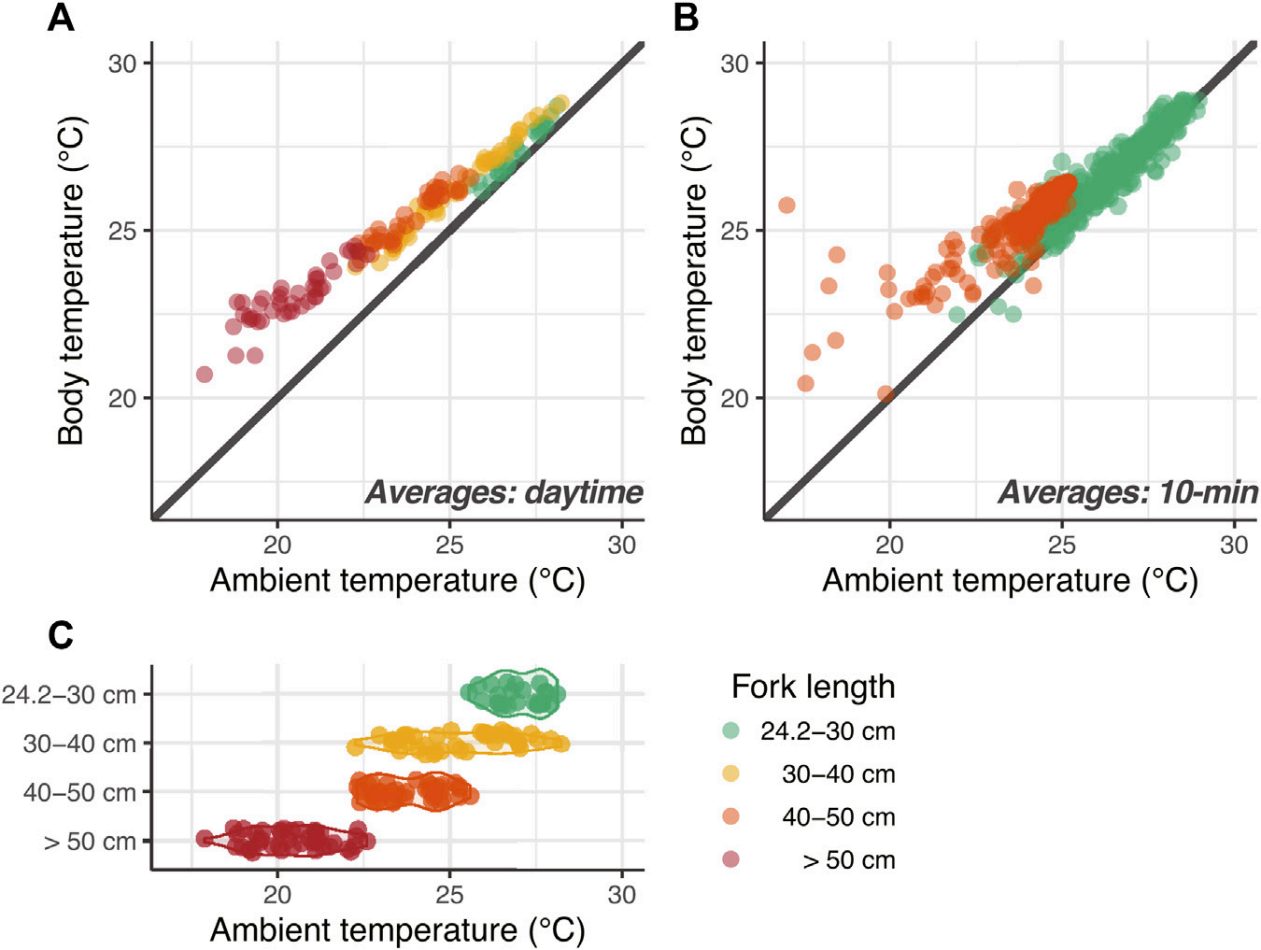
Thermal profiles of juvenile PBT. **(A)** Relationship between daytime mean ambient and body temperatures. Color indicates body size class. **(B)** Daytime mean ambient and body temperatures at 10 min intervals at 24 cm FL (green) and 45 cm FL (orange). **(C)** Daytime mean ambient temperature in each size class.

For the same individual, the average *T*_*b*_ and *T*_*a*_ during the daytime of each day indicate that *T*_*b*_ was maintained at 0.36 ± 1.12°C above *T*_*a*_ at 24.2–30 cm FL, but at 0.86 ± 1.27, 1.29 ± 0.8, and 2.00 ± 0.94°C at 30–40, 40–50, and 50–60 cm FL, respectively (Figure 3). Thus, the temperature difference tended to increase with growth (Figure 3A). While fish with 24.2–30 cm FL experienced *T*_*a*_ of 25.6–28.1°C, fish with 30–40 cm FL experienced a wider range of *T*_*a*_, and fish with FL ≥ 40 cm experienced a lower *T*_*a*_ of 17.9–25.6°C (Figures 3A,C).

In addition, we examined the relationship between daytime *T*_*b*_ and *T*_*a*_ every 10 min of an individual relative to its 24 and 45 cm FL, where the ability to maintain *T*_*b*_ was confirmed (Figure 3B). This demonstrated that the temperature difference increased with growth in the overlapping *T*_*a*_ range of 24–26°C. In the 24 cm FL range, *T*_*b*_ also decreased as *T*_*a*_ decreased from 29.0 to 22.0°C. However, in the 45 cm FL range, the difference between *T*_*b*_ and *T*_*a*_ increased as *T*_*a*_ decreased from 25.2 to 17.0°C. This suggests that *T*_*b*_ could be maintained by a mechanism on a time scale of approximately 10 min.

### 3.2 Changes in body heat insulation and heat production rate in pacific bluefin tuna that can maintain *T*_*b*_

**FIGURE 4:**
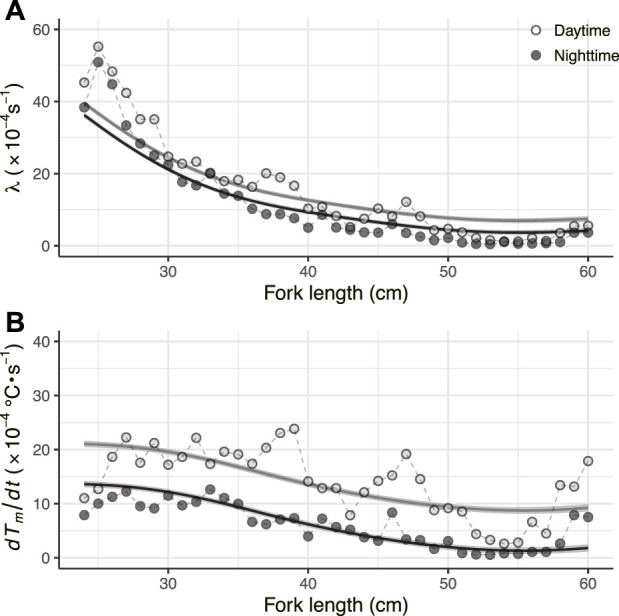
**(A)** Relationship between fork length and λ **(B)** Relationship between fork length and *dT*_*m*_ */dt*. Circles represent the parameters estimated semi-daily by the heat-budget model. Pale and dark gray colors indicate the data obtained in the daytime and nighttime, respectively. The smooth lines represent the fitted lines for ID 2012-0925 predicted by the generalized additive mixed model.

**FIGURE 5:**
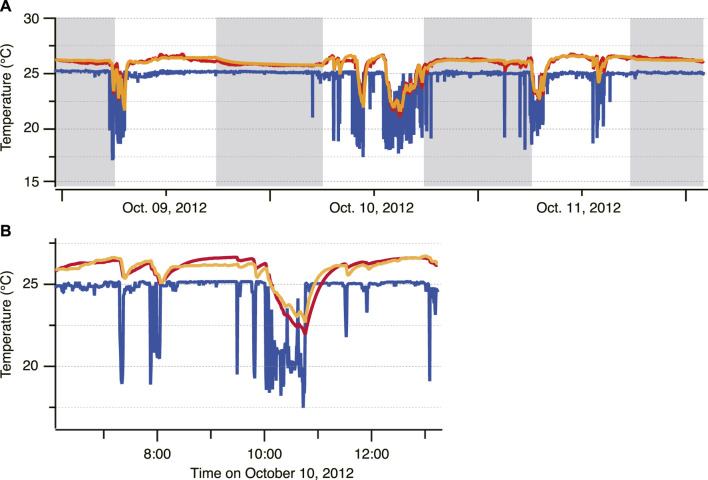
Time-series of the ambient temperature (blue) and body temperature (actual: red, predicted: orange) for ID 2012-0925 **(A)** and the enlarged data **(B)**.

For fish ID 2012-0925, the whole-body heat-transfer coefficient (*λ*) and heat production rate (*dT*_*m*_ */dt*) were calculated for day and night (Figures 4A,B). For verification, the predicted *T*_*b*_ was obtained by substituting *λ*, *dT*_*m*_ */dt*, and the measured *T*_*a*_ data into Eq. 1 and comparing the result with the actual *T*_*b*_ (Figure 5). The predicted and measured *T*_*b*_ were generally in agreement. Regarding *λ*, the average for 1 week from the start of measurement (24.2 cm FL) was 4.7 × 10^−3^ s^−1^, whereas the average for the week before individuals reached 45 cm FL was 5.6 × 10^−4^ s^−1^. This indicated that the *λ* of PBT decreased significantly with growth (Figure 4A). Regarding *dT*_*m*_ */dt*, the average for 1 week from the start of measurement (24.2 cm FL) was 1.0 × 10^−3^°C·s^−1^, and the average for the week before reaching 45 cm FL was 7.7 × 10^−4^°C s^−1^. Thus, *dT*_*m*_ */dt* decreased with growth, but its rate of decrease was slower than that of *λ*. In addition, *dT*_*m*_ */dt* varied more than *λ* (Figure 4B). Values of *λ* and *dT*_*m*_ */dt* in the daytime were higher than those in the nighttime throughout the measurement period. To evaluate the difference in the parameters between daytime and nighttime, the effects of FL on *λ* and *dT*_*m*_ */dt* were modeled in GAMMs as smoothing splines with all 41 fish. The models including the effects of day/night were selected for both parameters (Figure 4; Table 2).

**TABLE 2:** Effect of day/night on the heat-budget model parameters (λ and *dT*_*m*_ */dt*) using the generalized additive mixed model. The best-fitting models are indicated in bold. Random effect was modeled as the intercept.

Response variable	Model	AIC	BIC
λ	Null model		
	λ ∼ (intercept)	7434.5	7622.1
	Model 1		
	λ ∼ spline (*FL*)	6084.4	6310.3
	**Model 2**		
	**λ ∼ spline(*FL*) + Day/Night**	**5938.7**	**6172.0**
*dT*_*m*_ */dt*	Null model		
	*dT*_*m*_ */dt* ∼ (intercept)	6868.4	6878.2
	Model 1		
	*dT*_*m*_ */dt* ∼ spline (*FL*)	6379.3	6541.5
	**Model 2**		
	***dT***_***m***_ ***/dt* ∼ spline(*FL*) + Day/Night**	**5661.8**	**5863.3**

**FIGURE 6:**
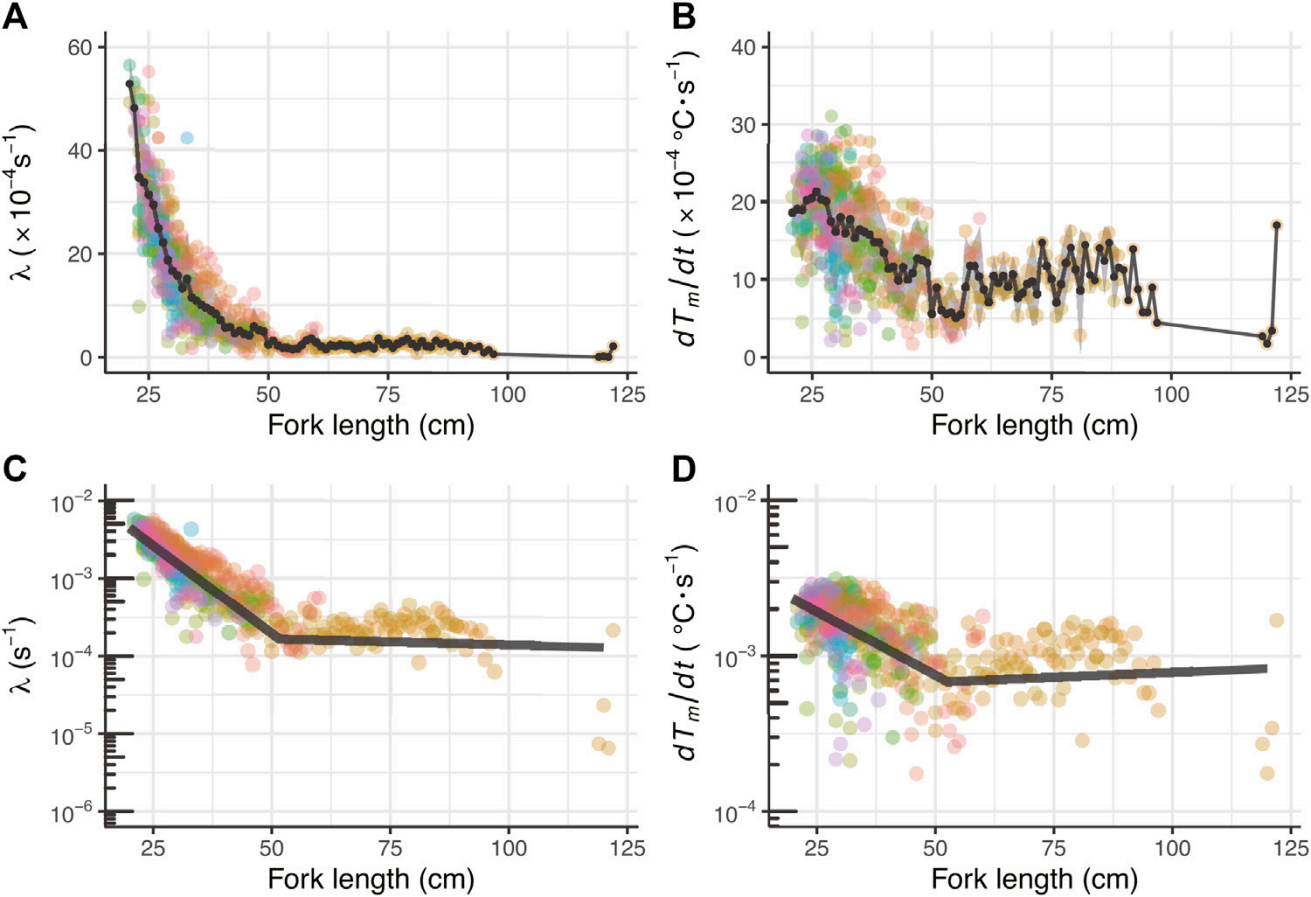
Changing relationship of *λ* and *dT*_*m*_ */dt* throughout their ontogeny **(A)** Relationship between fork length and λ **(B)** Relationship between fork length *dT*_*m*_ */dt*. The colored circles represent parameters estimated by the heat-budget model for each individual. The black circles connected by lines and the shaded area represent mean values and s.d. Per by fork length, respectively **(C)** Segment regression analysis for *λ* and **(D)** *dT*_*m*_ */dt*. The dark gray lines refer to the segment regression predicted lines for the log-transformed parameters.

The average and standard deviation values of the heat budget model’s parameters in the daytime (*λ* and *dT*_*m*_ */dt*) for all 41 fish are shown for each body size in Figures 6A,B; *λ* was 47.6 ± 4.3 × 10^−4^ s^−1^ at 21 cm FL. In contrast, when the FL was 45 cm, *λ* was 3.2 ± 1.9 × 10^−4^ s^−1^. Therefore, when the FL approximately doubled, *λ* greatly decreased, and the value became one order of magnitude smaller. Consequently, the large decrease in *λ* with growth was seen in individual ID 2012-0925 as well as in all 41 individuals. Since *dT*_*m*_ */dt* was 15.7 ± 1.4 × 10^−4^ s^−1^ at 21 cm FL and 6.3 ± 2.4 × 10^−4^ (s^−1^) at 45 cm FL, it gradually decreased with growth, a tendency observed in all individuals. In addition, as a result of segment regression, *λ* decreased rapidly to 52 cm FL (Figure 6C; Table 3). The rate of decrease in *dT*_*m*_ */dt* also changed above 52 cm FL (Figure 6D; Table 3).

**TABLE 3:** The relationship between heat-budget model parameters (λ and *dT*_*m*_ */dt*) and fork length modeled with mixed effect model with/without segmented regression. The best-fitting models are indicated in bold. Random effect was modeled as the intercept.

Response variable	Model	Break point (FL in cm)	AIC	BIC
λ	lme model		57.4	74.4
	**segmented model**	**51.6 (49.8–53.4)**	**−282.6**	**−257.1**
*dT*_*m*_ */dt*	lme model		**−**195.9	**−**178.9
	**segmented model**	**52.6 (47.8–57.3)**	**−268.8**	**−243.3**

## 4 Discussion

In ectothermic fish, *T*_*b*_ is approximately the same as *T*_*a*_ (e.g., salmonid fish [*Oncorhynchus keta*]; Azumaya and Ishida, 2005). In this study, in the data of the 20 PBT cm FL group whose *T*_*b*_ and *T*_*a*_ were measured in the field, there was no significant difference between *T*_*b*_ and *T*_*a*_ immediately after release. However, *T*_*b*_ tended to exceed *T*_*a*_ as the fish grew after measurement and recording (Figures 3A,B). In addition, the temperature difference increased in cooler water (see *T*_*a*_ for >50 cm FL in Figure 3). This suggests that this species can maintain *T*_*b*_, even in cooler oceanographic environments, and that the ability of this species to maintain *T*_*b*_ at the 20 cm FL stage had not yet developed but instead, developed as the fish grew.

### 4.1 Changes in body heat insulation and heat production rate in pacific bluefin tuna that can maintain *T*_*b*_

The predicted *T*_*b*_ was obtained for verification by substituting *λ*, *dT*_*m*_ */dt* and the measured *T*_*a*_ data into Eq. 1 and comparing the result with the actual *T*_*b*_ (Figure 5). The predicted and measured *T*_*b*_ were generally in agreement, and we considered that *λ* and *dT*_*m*_ */dt* did not include large errors.

Here, let us consider the meaning of this temperature difference by taking the average value of the temperature difference as the equilibrium state. Then, considering *T*_*b*_ as an equilibrium state; that is, the state where the left side of Eq. 1 is 0, Eq. 6 holds:

$$T_{b}-T_{a}=\frac{dT_{m}/dt}{\lambda },$$ (6)

meaning that the temperature difference between *T*_*b*_ and *T*_*a*_ is proportional to the heat production rate (*dT*_*m*_ */dt*) and increases in inverse proportion to the whole-body heat-transfer coefficient (*λ*). On the right-hand side of Eq. 6, when the PBT approximately doubled in size from 21 to 45 cm FL, the *dT*_*m*_ */dt* (numerator) was about 0.4 times lower, and the *λ* (denominator) was about 0.07 times lower, which was one order of magnitude lower than the numerator (Figures 6A,B). Therefore, the temperature difference on the left side increased with growth. This indicates that the decrease in *λ*—the improvement in the heat insulation of the fish body—has a strong influence on the development of PBT’s ability to maintain *T*_*b*_ in PBT.

To understand the rapid decrease in *λ* from 21 to 45 cm FL, the *λ* characteristics of fish were considered using the reciprocal of the heat time constant (1/*λ*). The thermal time constant is a constant that expresses the degree of responsiveness to temperature changes and refers to the time required for *T*_*b*_ to change to 1/e (base of the natural logarithm) (63.2%) of a specific temperature. Using this thermal time constant, the ability of PBT with 45–78 cm FL to maintain *T*_*b*_ with vertical movement has been discussed (Kitagawa et al., 2007b). When calculating the thermal time constant of the data obtained in this study, it took 3.5, 19.0, and 51.0 min for PBT with 21, 36, and 45 cm FL to change, respectively, indicating that it takes longer for *T*_*b*_ to change with growth. This finding indicates that, as the body grows, *T*_*b*_ becomes less affected by *T*_*a*_. This is consistent with the fact that in terms of the relationship between *T*_*b*_ and *T*_*a*_ every 10 min shown in Figure 4B, *T*_*b*_ at 45 cm FL was less susceptible to a *T*_*a*_ decrease than at FL 21 cm. Moreover, this is consistent with both the present result that showed *λ* rapidly decreases up to the breakpoint found at approximately 52 cm FL (Figure 6) and the previous report that showed the time of individuals of this species with 45–78 cm FL spent below the thermocline was approximately 5–10 min (Kitagawa et al., 2007b).

**FIGURE 7:**
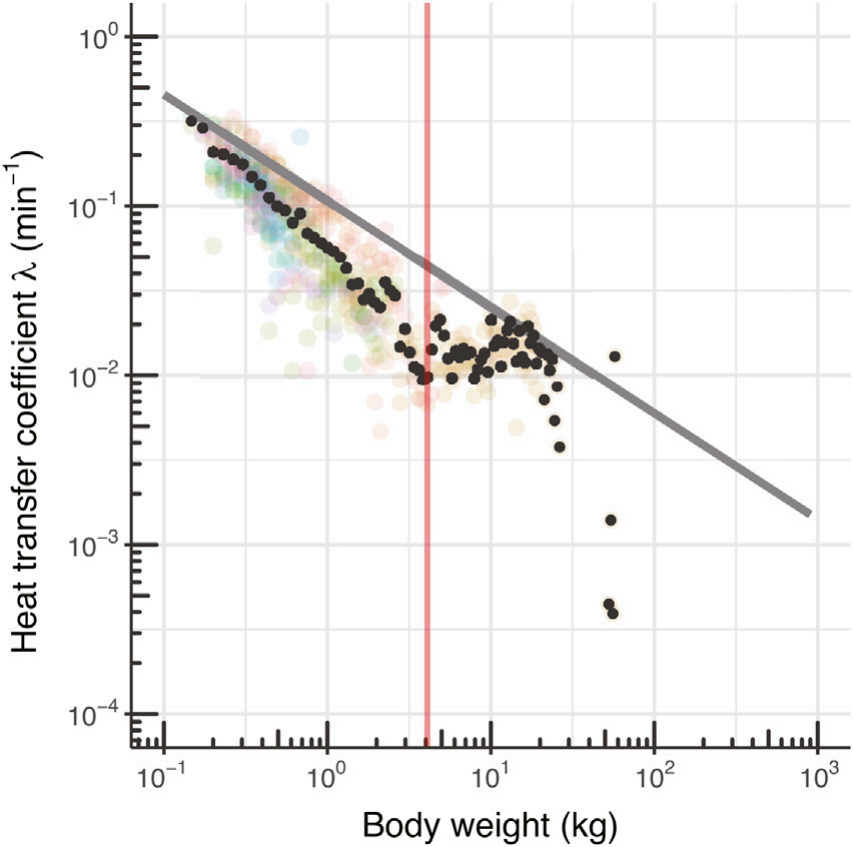
Relationship between body mass and heat-transfer coefficients *λ* of juvenile PBT. Black-filled circles and colored transparent circles represent the mean value and each individual value of λ, respectively. The red vertical line indicates the breakpoint of body size (52 cm) of *λ* in Figure 5. The body-size scaling of *λ* estimated by a previous study (Nakamura et al., 2020) is indicated by the bold gray line.

According to a study comparing the *λ* of 19 species, including PBT, alewife (*Alosa pseudoharengus*; approximately 30 g), and whale shark (*Rhincodon typus*; approximately 1,600 kg) (Nakamura et al., 2020), larger individuals tended to have a lower *λ* due to their increased heat capacity (Neill et al., 1974; Schmidt-Nielsen, 1984; Nakamura et al., 2020) (Figure 7). Meanwhile, the *λ* of PBT in this study rapidly decreased (up to 52 cm FL), which cannot be explained solely by the increase in heat capacity due to the increase in body size and difference in flesh quality (Figure 7). The *T*_*b*_ conservation organ in PBT consists of two types of rete mirabile, a lateral net that develops to cover the red muscles, which constitute a heat source, and a visceral net distributed on the surface of the liver (Sharp and Dizon, 1978; Stevens and Neill, 1978; Malik et al., 2020; Malik et al., 2021). The length of the lateral net, number of blood vessels, and cross-sectional area of the visceral net also rapidly increase in PBT as the FL increases from 18.5 to 62.5 cm (Malik et al., 2020). Accordingly, in addition to the rapid increase in heat capacity, the rapid development of lateral nets is a factor in improving the heat-insulating properties of PBT (Funakoshi et al., 1985; Kubo et al., 2008; Malik et al., 2020; Malik et al., 2021). Aside from the increase in body size, additionally, white muscle also developed to cover red muscle to increase the distance from red muscle mass to the body’s surface (red muscle internalization: Graham and Dickson, 2001), thereby reducing the temperature gradient (Kitagawa et al., 2006).

In this study, the *λ* values in the daytime were significantly higher than those at night. Kitagawa et al. (2001), Kitagawa et al. (2006), Kitagawa et al. (2007b) investigated *λ* for young PBT but estimated the values quite approximately and considered the difference between daytime and nighttime to be minor. Therefore, the difference presented here, i.e., higher in the daytime according to highly resolved, *in situ* sensing, is a new observation, although Fujioka et al. (2021) reported that *λ* for adult fish increased when they experienced warm surface waters, probably to avoid overheating at night. In previous studies, bigeye tuna (*T. obesus*: BET), widely inhabiting temperate to tropical areas, was thought to modulate *T*_*b*_ by changing the function of the rete mirabile to regulate cardiac output (Holland et al., 1992; Malte et al., 2007; Hino et al., 2021). This suggests that the cardiac output probably actively increased, and the rete mirabile function was reduced by more activity individuals during the daytime.

The values of *dT*_*m*_ */dt* also decreased, albeit moderately, with growth (Figure 6B). This may be because the amount of heat produced per unit of body weight decreases with growth (Kitagawa et al., 2006). The decrease in calorific value with growth may be related to the corresponding decreases in metabolic rate (Gillooly et al., 2001; Yagi et al., 2010; Gavrilov, 2013) and relative red muscle mass (Malik et al., 2020). The difference in *dT*_*m*_ */dt* between daytime and nighttime may be because the amount of movement of PBT, such as the frequency of vertical movement associated with feeding behavior, changes between the day and night (Kitagawa et al., 2000; Kitagawa et al., 2004).

### 4.2 Adaptation of pacific bluefin tuna to temperate waters

There are eight species of tuna (genus *Thunnus*). PBT, ABT, SBT, and albacore (*T. alalunga*) mainly distributes in temperate waters. By contrast, yellowfin tuna (YFT), longtail tuna (*T. tonggol*), and blackfin tuna (*T. atlanticus*) are mainly found in subtropical to tropical waters, and BET are distributed in waters between 50ºN and 45ºS (ICCAT, 2019). Comparing the *λ* values of PBT with those of YFT, BET, and salmon (Azumaya and Ishida, 2005; Kitagawa et al., 2006; Kubo et al., 2008; Aoki et al., 2020; Hino et al., 2021), the value for PBT was not only smaller than those of salmon, which is an exothermic species, but also YFT and BET. This suggests that the heat-insulating properties of the fish are better than those of other species.

The term λ consists of two elements and is described as in Eq. 7 (Kitagawa and Kimura, 2006; Kitagawa et al., 2007b; Aoki et al., 2020; Hino et al., 2021).

λ=k+w, (7)

where *k* indicates the physical effect (s^−1^) of the fish body (thermal inertia) and *w* indicates the physiological effect of the heat exchange between the body and the environment, which is largely determined by physiological mechanisms, such as control of blood flow (s^−1^). The decrease in *w* is thought to be caused by a decrease in cardiac output or promotion of heat exchange in the rete mirabile (Holland et al., 1992; Kitagawa and Kimura, 2006; Kitagawa et al., 2007b; Aoki et al., 2020). There is little difference in the *k* values of PBT (personal observation) and YFT in the 40 cm FL range (4.5 × 10^–4^ to 8.4 × 10^–4^ s^−1^; Aoki et al., 2020). Therefore, the difference in the magnitude of *λ* is considered to be due to the development of the mirabile and the difference in its function, as the lateral net of PBT is more developed than that of YFT (Sharp and Dizon, 1978; Stevens and Neill, 1978). The values of λ indicate that PBT have a more advanced rete mirabile as they can adapt to temperate waters at higher latitudes than tropical waters. As a side note, there are additional physiological adaptations regarding blood oxygen binding (e.g. Dickson, 1996; Lilly et al., 2015) that support function in *T*_*a*_ down to less than 5°C for very short periods, as confirmed for ABT (Block et al., 2001).

PBT breed from April to June in spawning grounds between the Philippines and the Ryukyu Islands of Japan and during August in the Sea of Japan (Okiyama, 1974; Chen et al., 2006; Tanaka et al., 2007) after being transported by sea currents to Japanese coastal areas in the summer (e.g., by the Kuroshio Current) 60–90 days after hatching (Tanaka et al., 2006; Kitagawa et al., 2010). As Kitagawa et al. (2010) discussed, they may spawn during this time because Japanese coastal waters have only a limited period of favorable water temperatures for juvenile and young growth. The ambient temperatures around the nursery are only favorable for growth during August and September (Kitagawa et al., 2010). After moving to Japanese coastal areas and reaching an FL of 25 cm, PBT rapidly develop swimming abilities and switch from a diet of small squid and zooplankton to a diet based on fish prey items that have greater body mass with higher calorific content and greater swimming ability, such as epipelagic fish species distributed in temperate waters (*Etrumeus teres, Sardinops melanostictus,* and *Engraulis japonicus*; Shimose et al., 2013; Kitagawa and Fujioka, 2017).

PBT make repeated short-duration dives across the thermocline in coastal areas, probably for feeding (Furukawa et al., 2017). Extensive vertical movement exposes the fish to marked changes in *T*_*a*_, particularly fish with FL < ca. 40 cm (before October), which are easily affected by low *T*_*a*_ (Figures 2, 3). Therefore, they must move back and forth between the warm surface layer and the thermocline to recover the *T*_*b*_ lowered by vertical movement (Furukawa et al., 2017). When they reach 52 cm FL or more, their body-heat conservation ability significantly increases as their body insulation has improved as clarified in the present study. Thus, they can stay close to the thermocline for a long time and actively feed on the more abundant prey organisms occurring there. Furthermore, by maintaining *T*_*b*_ significantly above *T*_*a*_ and acquiring low-temperature resistance, especially for the heart (cardiac output) (Blank et al., 2004), they can withstand the subsequent seasonal drop in *T*_*a*_ from autumn to winter, which enables overwintering. In the next year, they can move to the Kuroshio-Oyashio transition region (Figure 1), located further north of Tosa Bay, where prey organisms are abundant at the surface layer and achieve further growth (Kitagawa et al., 2004).

This study was conducted to determine at what size PBT develop their thermo-conservation ability by monitoring (i.e., physio-logging) the *T*_*b*_ and *T*_*a*_ of free-swimming juveniles in nature as they grow, although some previous laboratory studies investigated (Funakoshi et al., 1985; Kubo et al., 2008; Malik et al., 2020; Malik et al., 2021). This study quantified their heat-insulation property (whole-body heat-transfer coefficient: *λ*) and internal heat production (the heat production rate: *dT*_*m*_ */dt*). Juvenile PBT rapidly acquire higher thermo-conservation ability, including thermal insulation, together with swimming abilities and diet-switching after they move to Japanese coastal waters. This suggests that the trait is the result of an adaptation allowing the fish to capture high-quality prey abundant in temperate waters to achieve a high growth rate during the yearling period. Also, this species exhibits better thermo-conservation ability than other *Thunnus* species inhabiting tropical/subtropical zones, providing a selective advantage and allow high energetic expenditure with high oxygen demand (Brill, 1987; Brill, 1996; Blank et al., 2002; Blank et al., 2007). To elucidate their ontogenetic shift in temperate waters of decreasing ambient temperature more precisely, changes in their physiological capacity, such as the oxygen consumption rate or myoglobin content in muscle, should also be investigated. Furthermore, recent global warming brings an increase in ocean temperatures and could change the migration route and distribution of PBT in the future. By continuing to monitor *T*_*b*_ and *T*_*a*_ for PBT, the energy budget for their growth could be clarified, which may help forecast their future distribution for resource sustainability and management purposes.

## Data Availability

The raw data supporting the conclusions of this article will be made available by the authors upon reasonable request.

## References

[B1] Altringham J. D. Block B. A. (1997). Why do tuna maintain elevated slow muscle temperatures? Power output of muscle isolated from endothermic and ectothermic fish. J. Exp. Biol. 200, 2617–2627. 10.1242/jeb.200.20.2617 9359368

[B2] Aoki Y. Aoki A. Ohta I. Kitagawa T. (2020). Physiological and behavioural thermoregulation of juvenile yellowfin tuna *Thunnus albacares* in subtropical waters. Mar. Biol. 167, 71–14. 10.1007/s00227-020-03679-w

[B3] Azumaya T. Ishida Y. (2005). Mechanism of body cavity temperature regulation of chum salmon (*Oncorhynchus keta*) during homing migration in the North Pacific Ocean. Fish. Oceanogr. 14, 81–96. 10.1111/j.1365-2419.2004.00323.x

[B4] W H Bayliff (Editor) (1980). Synopses of biological data on eight species of scombrids (La Jolla, California: Inter-American Tropical Tuna Commission). Special Report No. 2.

[B5] Bayliff W. H. Ishizuki Y. Deriso R. B. (1991). Growth, movement, and attrition of northern bluefin tuna, *Thunnus thynnus*, in the Pacific Ocean, as determined by tagging. Inter-American Trop. Tuna Comm. Bull. 20, 1–94.

[B6] Bernal D. Brill R. W. Dickson K. A. Shiels H. A. (2017). Sharing the water column: Physiological mechanisms underlying species-specific habitat use in tunas. Rev. Fish. Biol. Fish. 27, 843–880. 10.1007/s11160-017-9497-7

[B7] Blank J. M. Farwell C. J. Morrissette J. M. Schallert R. J. Block B. A. (2007). Influence of swimming speed on metabolic rates of juvenile Pacific bluefin tuna and yellowfin tuna. Physiol. Biochem. Zool. 80, 167–177. 10.1086/510637 17252513

[B8] Blank J. M. Morrissette J. M. Davie P. S. Block B. A. (2002). Effects of temperature, epinephrine and Ca2+ on the hearts of yellowfin tuna (*Thunnus albacares*). J. Exp. Biol. 205, 1881–1888. 10.1242/jeb.205.13.1881 12077164

[B9] Blank J. M. Morrissette J. M. Landeira-Fernandez A. M. Blackwell S. B. Williams T. D. Block B. A. (2004). *In situ* cardiac performance of Pacific bluefin tuna hearts in response to acute temperature change. J. Exp. Biol. 207, 881–890. 10.1242/jeb.00820 14747418

[B10] Block B. A. Dewar H. Blackwell S. B. Williams T. D. Prince E. D. Farwell C. J. (2001). Migratory movements, depth preferences, and thermal biology of Atlantic bluefin tuna. Science 293, 1310–1314. 10.1126/science.1061197 11509729

[B11] Block B. A. Finnerty J. R. (1994). Endothermy in fishes: A phylogenetic analysis of constraints, predispositions, and selection pressures. Environ. Biol. Fishes 40, 283–302. 10.1007/BF00002518

[B12] Block B. A. (2005). Physiological ecology in the 21st century: Advancements in biologging science. Integr. Comp. Biol. 45, 305–320. 10.1093/icb/45.2.305 21676774

[B13] Boustany A. M. Matteson R. Castleton M. Farwell C. Block B. A. (2010). Movements of Pacific bluefin tuna (*Thunnus orientalis*) in the Eastern North Pacific revealed with archival tags. Prog. Oceanogr. 86, 94–104. 10.1016/j.pocean.2010.04.015

[B14] Boyd S. Vandenberghe L. (2018). Introduction to applied linear algebra: Vectors, matrices, and least squares. Cambridge: Cambridge University Press.

[B15] Brill R. W. (1987). On the standard metabolic rates of tropical tunas, including the effect of body size and acute temperature change. Fish. Bull. 85, 25–35.

[B16] Brill R. W. (1996). Selective advantages conferred by the high performance physiology of tunas, billfishes, and dolphin fish. Comp. Biochem. Physiology Part A Physiology 113, 3–15. 10.1016/0300-9629(95)02064-0

[B17] Carey F. G. (1973). Fishes with warm bodies. Sci. Am. 228, 36–44. 10.1038/scientificamerican0273-36 4684579

[B18] Carey F. G. Gibson Q. H. (1983). Heat and oxygen exchange in the rete mirabile of the bluefin tuna, *Thunnus thynnus* . Comp. Biochem. Physiology Part A Physiology 74, 333–342. 10.1016/0300-9629(83)90612-6

[B19] Carey F. G. Kanwisher J. W. Stevens E. D. (1984). Bluefin tuna warm their viscera during digestion. J. Exp. Biol. 109, 1–20. 10.1242/jeb.109.1.1

[B20] Carey F. G. Teal J. M. (1966). Heat conservation in tuna fish muscle. Proc. Natl. Acad. Sci. U. S. A. 56, 1464–1469. 10.1073/pnas.56.5.1464 16591392 PMC220001

[B21] Carey F. G. Teal J. M. Kanwisher J. W. Lawson K. D. Beckett J. S. (1971). Warm-bodied fish. Am. Zool. 11, 137–143. 10.1093/icb/11.1.137

[B22] Carey F. G. (1981). “Warm fish,” in A companion to animal Physiology. Editors Taylor C. R. Johansen K. Bolis L. (Cambridge: Cambridge University Press), 216–233.

[B23] Chen K. S. Crone P. Hsu C. C. (2006). Reproductive biology of female Pacific bluefin tuna *Thunnus orientalis* from south-Western North Pacific Ocean. Fish. Sci. 72, 985–994. 10.1111/j.1444-2906.2006.01247.x

[B24] Clemens A. Fittner G. (1969). Bluefin tuna migrate across the Pacific Ocean. Calif. Fish. Game 55, 132–135.

[B25] Dickson K. A. Graham J. B. (2004). Evolution and consequences of endothermy in fishes. Physiol. Biochem. Zool. 77, 998–1018. 10.1086/423743 15674772

[B26] Dickson K. A. (1996). Locomotor muscle of high-performance fishes: What do comparisons of tunas with ectothermic sister taxa reveal? Comp. Biochem. Physiology Part A Physiology 113, 39–49. 10.1016/0300-9629(95)02056-X

[B27] Eschricht D. Müller J. (1835). Uber die arteriösen and venösen wundernetz an der leber und einen merkwürdigen bau dieses organes beim thunfische. Abh. Dtsch. Akad. Wiss. Berl., 1–30.

[B28] Fudge D. S. Stevens E. D. (1996). The visceral retia mirabilia of tuna and sharks: An annotated translation and discussion of the eschricht and müller 1835 paper and related papers. Guelph Ichthyol. Rev. 4, 1–92.

[B29] Fujioka K. Fukuda H. Furukawa S. Tei Y. Okamoto S. Ohshimo S. (2018). Habitat use and movement patterns of small (age-0) juvenile Pacific bluefin tuna (*Thunnus orientalis*) relative to the Kuroshio. Fish. Oceanogr. 27, 185–198. 10.1111/fog.12244

[B30] Fujioka K. Sasagawa K. Kuwahara T. Estess E. E. Takahara Y. Komeyama K. (2021). Habitat use of adult pacific bluefin tuna *Thunnus orientalis* during the spawning season in the Sea of Japan: Evidence for a trade-off between thermal preference and reproductive activity. Mar. Ecol. Prog. Ser. 668, 1–20. 10.3354/meps13746

[B31] Fukuda H. Uyama H. Oshima K. (2015). A minor change in the estimation of length composition data of Japanese troll fisheries. Kaohsiung, Taiwan: International Scientific Committee for Tuna and Tuna-Like Species in the North Pacific Ocean (ISC). ISC/15/PBFWG-2/03.

[B32] Funakoshi S. Wada K. Suzuki T. (1985). Development of the rete mirabile with growth and muscle temperature in the young bluefin tuna. Bull. Jpn. Soc. Sci. Fish. 51, 1971–1975. 10.2331/suisan.51.1971

[B33] Furukawa S. Fujioka K. Fukuda H. Suzuki N. Tei Y. Ohshimo S. (2017). Archival tagging reveals swimming depth and ambient and peritoneal cavity temperature in age-0 Pacific bluefin tuna, *Thunnus orientalis*, off the southern coast of Japan. Environ. Biol. Fishes 100, 35–48. 10.1007/s10641-016-0552-3

[B34] Gavrilov V. M. (2013). Origin and development of homoiothermy: A case study of avian energetics. Adv. Biosci. Biotechnol. 4, 1–17. 10.4236/abb.2013.48A1001 24883227

[B35] Gibbs R. H. Jr Collette B. B. (1967). Comparative anatomy and systematics of the tunas, genus *Thunnus* . Fish. Bull. 66, 65–130.

[B36] Gillooly J. F. Brown J. H. West G. B. Savage V. M. Charnov E. L. (2001). Effects of size and temperature on metabolic rate. Science 293, 2248–2251. 10.1126/science.1061967 11567137

[B37] Graham J. B. Dickson K. A. (2001). “Anatomical and physiological specializations for endothermy,” in Tuna: Physiology, ecology, and evolution. Editors Block B. A. Stevens E. D. (San Diego: Academic Press), 121–165.

[B38] Graham J. B. (1975). Heat exchange in the yellowfin tuna, *Thunnus albacares*, and skipjack tuna, *Katsuwonus pelamis*, and the adaptive significance of elevated body temperatures in scombrid fishes. Fish. Bull. 73, 219–229.

[B39] Hino H. Kitagawa T. Matsumoto T. Aoki Y. Kimura S. (2021). Development of behavioral and physiological thermoregulatory mechanisms with body size in juvenile bigeye tuna *Thunnus obesus* . Fish. Oceanogr. 30, 219–231. 10.1111/fog.12515

[B40] Holland K. N. Brill R. W. Chang R. K. Sibert J. R. Fournier D. A. (1992). Physiological and behavioural thermoregulation in bigeye tuna (*Thunnus obesus*). Nature 358, 410–412. 10.1038/358410a0 1641023

[B41] ICCAT (2019). Report of the standing committee on research and statistics (SCRS). Madrid, Spain: ICCAT, 44–61.

[B42] Inagake D. Yamada H. Segawa K. Okazaki M. Nitta A. Itoh T. (2001). Migration of young bluefin tuna, *Thunnus orientalis* Temminck et Schlegel, through archival tagging experiments and its relation with oceanographic conditions in the Western North Pacific. Bull. Natl. Res. Inst. Far Seas. Fish. 38, 53–81.

[B43] Itoh T. Tsuji S. Nitta A. (2003). Migration patterns of young Pacific bluefin tuna (*Thunnus orientalis*) determined with archival tags. Fish. Bull. 101, 514–534.

[B44] Jusup M. Klanjscek T. Matsuda H. Kooijman S. (2011). A full lifecycle bioenergetic model for bluefin tuna. PLoS One 6, e21903. 10.1371/journal.pone.0021903 21779352 PMC3133599

[B45] Kishinouye K. (1923). Contribution to the comparative study of the so-called scombrid fishes. J. Coll. Agric. Imp. Univ. Tokyo 8, 295–475.

[B46] Kitagawa T. (2013). “Behavioral ecology and thermal physiology of imature Pacific bluefin tuna,” in Physiology and ecology of fish migration. Editors Ueda H. Tsukamoto K. (Boca Raton: CRC Press), 152–178.

[B47] Kitagawa T. Boustany A. M. Farwell C. J. Williams T. D. Castleton M. R. Block B. A. (2007a). Horizontal and vertical movements of juvenile bluefin tuna (*Thunnus orientalis*) in relation to seasons and oceanographic conditions in the eastern Pacific Ocean. Fish. Oceanogr. 16, 409–421. 10.1111/j.1365-2419.2007.00441.x

[B48] Kitagawa T. Fujioka K. (2017). Rapid ontogenetic shift in juvenile Pacific bluefin tuna diet. Mar. Ecol. Prog. Ser. 571, 253–257. 10.3354/meps12129

[B49] Kitagawa T. Fujioka K. Suzuki N. (2019). “Electronic tagging applications and migrations of Pacific bluefin tuna in the Western Pacific Ocean,” in The future of bluefin tunas: Ecology, fisheries management, and conservation. Editor Block B. A. (Baltimore: Johons Hopkins Univ. Press), 147–164.

[B50] Kitagawa T. Kato Y. Miller M. J. Sasai Y. Sasaki H. Kimura S. (2010). The restricted spawning area and season of pacific bluefin tuna facilitate use of nursery areas: A modeling approach to larval and juvenile dispersal processes. J. Exp. Mar. Biol. Ecol. 393, 23–31. 10.1016/j.jembe.2010.06.016

[B51] Kitagawa T. Kimura S. (2006). An alternative heat-budget model relevant to heat transfer in fishes and its practical use for detecting their physiological thermoregulation. Zool. Sci. 23, 1065–1071. 10.2108/zsj.23.1065 17261919

[B52] Kitagawa T. Kimura S. Nakata H. Yamada H. (2004). Diving behavior of immature, feeding pacific bluefin tuna (*Thunnus thynnus orientalis*) in relation to season and area: the east China sea and the kuroshio–oyashio transition region. Fish. Oceanogr. 13, 161–180. 10.1111/j.1365-2419.2004.00282.x

[B53] Kitagawa T. Kimura S. Nakata H. Yamada H. Nitta A. Sasai Y. (2009). Immature Pacific bluefin tuna, *Thunnus orientalis*, utilizes cold waters in the Subarctic Frontal Zone for trans-Pacific migration. Environ. Biol. Fishes 84, 193–196. 10.1007/s10641-008-9409-8

[B54] Kitagawa T. Kimura S. Nakata H. Yamada H. (2006). Thermal adaptation of Pacific bluefin tuna *Thunnus orientalis* to temperate waters. Fish. Sci. 72, 149–156. 10.1111/j.1444-2906.2006.01129.x

[B55] Kitagawa T. Kimura S. Nakata H. Yamada H. (2007b). Why do young Pacific bluefin tuna repeatedly dive to depths though the thermocline? Fish. Sci. 73, 98–106. 10.1111/j.1444-2906.2007.01307.x

[B56] Kitagawa T. Nakata H. Kimura S. Itoh T. Tsuji S. Nitta A. (2000). Effect of ambient temperature on the vertical distribution and movement of Pacific bluefin tuna *Thunnus thynnus orientalis* . Mar. Ecol. Prog. Ser. 206, 251–260. 10.3354/meps206251

[B57] Kitagawa T. Nakata H. Kimura S. Tsuji S. (2001). Thermoconservation mechanisms inferred from peritoneal cavity temperature in free-swimming Pacific bluefin tuna *Thunnus thynnus orientalis* . Mar. Ecol. Prog. Ser. 220, 253–263. 10.3354/meps220253

[B58] Kubo T. Sakamoto W. Murata O. Kumai H. (2008). Whole-body heat transfer coefficient and body temperature change of juvenile Pacific bluefin tuna *Thunnus orientalis* according to growth. Fish. Sci. 74, 995–1004. 10.1111/j.1444-2906.2008.01617.x

[B59] Lilly L. E. Bonaventura J. Lipnick M. S. Block B. A. (2015). Effect of temperature acclimation on red blood cell oxygen affinity in Pacific bluefin tuna (*Thunnus orientalis*) and yellowfin tuna (*Thunnus albacares*). Comp. Biochem. Physiol. A Mol. Integr. Physiol. 181, 36–44. 10.1016/j.cbpa.2014.11.014 25434601

[B60] Malik A. Dickson K. A. Kitagawa T. Fujioka K. Estess E. E. Farwell C. (2021). Scaling of locomotor muscle oxidative and glycolytic metabolic enzymes during the ontogeny of regional endothermy in Pacific bluefin tuna (*Thunnus orientalis*). Mar. Biol. 168, 130. 10.1007/s00227-021-03945-5

[B61] Malik A. Dickson K. A. Kitagawa T. Fujioka K. Estess E. E. Farwell C. (2020). Ontogeny of regional endothermy in Pacific bluefin tuna (*Thunnus orientalis*). Mar. Biol. 167, 133. 10.1007/s00227-020-03753-3

[B62] Malte H. Larsen C. Musyl M. Brill R. (2007). Differential heating and cooling rates in bigeye tuna (*Thunnus obesus* lowe): A model of non-steady state heat exchange. J. Exp. Biol. 210, 2618–2626. 10.1242/jeb.003855 17644676

[B63] Nakamura I. Matsumoto R. Sato K. (2020). Body temperature stability in the whale shark, the world’s largest fish. J. Exp. Biol. 223, jeb210286. 10.1242/jeb.210286 32366688

[B64] Neill W. H. Stevens E. D. Carey F. G. Lawson K. D. Mrosovsky N. Frair W. (1974). Letter: Thermal inertia versus thermoregulation in "warm" turtles and tunas. Science 184, 1008–1010. 10.1126/science.184.4140.1008 4826167

[B65] Okiyama M. (1974). Occurrence of the postlarvae of bluefin tuna, *Thunnus thynnus*, in the Japan Sea. Jpn. Sea Reg. Fish. Res. Lab. Bull. 25, 89–97. (in Japanese).

[B66] Orange C. J. Fink B. D. (1963). Migration of tagged bluefin tuna across the Pacific Ocean. Calif. Fish. Game 49, 307–309.

[B67] Sakamoto K. Q. Sato K. Ishizuka M. Watanuki Y. Takahashi A. Daunt F. (2009). Can ethograms be automatically generated using body acceleration data from free-ranging birds? PloS one 4, e5379. 10.1371/journal.pone.0005379 19404389 PMC2671159

[B68] Satoh K. (2010). Horizontal and vertical distribution of larvae of Pacific bluefin tuna *Thunnus orientalis* in patches entrained in mesoscale eddies. Mar. Ecol. Prog. Ser. 404, 227–240. 10.3354/meps08431

[B69] Satoh K. Tanaka Y. Iwahashi M. (2008). Variations in the instantaneous mortality rate between larval patches of Pacific bluefin tuna *Thunnus orientalis* in the northwestern Pacific Ocean. Fish. Res. 89, 248–256. 10.1016/j.fishres.2007.09.003

[B70] Schmidt-Nielsen K. (1997). Animal physiology: Adaptation and environment. 5th Edition. Cambridge: Cambridge University Press.

[B71] Schmidt-Nielsen K. (1984). Scaling: Why is animal size so important? Cambridge: Cambridge University Press.

[B72] Sharp G. D. Dizon A. E. (1978). “Color plates,” in The physiological ecology of tunas. Editors Sharp G. D. Dizon A. E. (New York: Academic Press), 15–24.

[B73] Shimose T. Watanabe H. Tanabe T. Kubodera T. (2013). Ontogenetic diet shift of age-0 year Pacific bluefin tuna *Thunnus orientalis* . J. Fish. Biol. 82, 263–276. 10.1111/j.1095-8649.2012.03483.x 23331149

[B74] Stevens E. D. Carey F. G. (1981). One why of the warmth of warm-bodied fish. Am. J. Physiol. 240, R151–R155. 10.1152/ajpregu.1981.240.3.R151 7212086

[B75] Stevens E. D. Neill W. H. (1978). “Body temperature relations of tunas, especially skipjack,” in Fish Physiology. Editors Hoar W. S. Randall D. J. (New York: Academic Press), 7, 316–359.

[B76] Tanaka Y. Mohri M. Yamada H. (2007). Distribution, growth and hatch date of juvenile Pacific bluefin tuna *Thunnus orientalis* in the coastal area of the Sea of Japan. Fish. Sci. 73, 534–542. 10.1111/j.1444-2906.2007.01365.x

[B77] Tanaka Y. Satoh K. Iwahashi M. Yamada H. (2006). Growth-dependent recruitment of Pacific bluefin tuna *Thunnus orientalis* in the northwestern Pacific Ocean. Mar. Ecol. Prog. Ser. 319, 225–235. 10.3354/meps319225

[B78] Tanaka Y. Tawa A. Ishihara T. Sawai E. Nakae M. Masujima M. (2019). Occurrence of pacific bluefin tuna *Thunnus orientalis* larvae off the pacific coast of tohoku area, northeastern Japan: Possibility of the discovery of the third spawning ground. Fish. Oceanogr. 29, 46–51. 10.1111/fog.12445

[B79] Watanabe Y. Y. Goldman K. J. Caselle J. E. Chapman D. D. Papastamatiou Y. P. (2015). Comparative analyses of animal-tracking data reveal ecological significance of endothermy in fishes. Proc. Natl. Acad. Sci. U. S. A. 112, 6104–6109. 10.1073/pnas.1500316112 25902489 PMC4434765

[B80] Whitlock R. Walli A. Cermeño P. Rodriguez L. Farwell C. Block B. (2013). Quantifying energy intake in Pacific bluefin tuna (*Thunnus orientalis*) using the heat increment of feeding. J. Exp. Biol. 216, 4109–4123. 10.1242/jeb.084335 24133153

[B81] Yagi M. Kanda T. Takeda T. Ishimatsu A. Oikawa S. (2010). Ontogenetic phase shifts in metabolism: Links to development and anti-predator adaptation. Proc. Biol. Sci. 277, 2793–2801. 10.1098/rspb.2010.0583 20444717 PMC2981994

